# Making a greener planet: nature documentaries promote plant awareness

**DOI:** 10.1093/aob/mcac149

**Published:** 2023-02-16

**Authors:** Joanna Kacprzyk, Stephanie Clune, Clare Clark, Adam Kane

**Affiliations:** School of Biology and Environmental Science, University College Dublin, Dublin 4, Ireland; School of Biology and Environmental Science, University College Dublin, Dublin 4, Ireland; School of Biology and Environmental Science, University College Dublin, Dublin 4, Ireland; School of Biology and Environmental Science, University College Dublin, Dublin 4, Ireland

**Keywords:** Plant conservation, plant blindness, plant awareness disparity, big data, David Attenborough, nature documentary, Wikipedia, Google Trends, science communication

## Abstract

**Background and Aims:**

Plants underpin life on Earth and are essential to human existence. Alarmingly, almost 40% of plant species are under threat of extinction, with plants that are not directly useful to humans being particularly vulnerable. Plant diversity and its untapped resources require urgent protection to safeguard our future, but conservation initiatives are biased towards mammals and birds. Plant awareness disparity, formerly known as plant blindness, describes our tendency to ignore plant life and has been suggested to play a crucial role in the bias against funding and support for plant conservation programmes. Previous studies indicate that nature documentaries can generate shifts in audience awareness of animal species by providing vicarious connections to nature. Here, we investigated whether the plant-focused popular BBC show *Green Planet* had a similar effect for plants and stimulated audience engagement for information after the broadcast.

**Methods:**

Online searches for further information were considered a form of engagement for evaluation of the interest of the audience in plants portrayed in *Green Planet* episodes. The big data activities (Google search engine and Wikipedia pageviews trends) related to the plants mentioned in *Green Planet* episodes were examined over the period covering the broadcast of the show in UK.

**Key Results:**

Analyses indicate that *Green Planet* generated increased awareness and stimulated audience engagement for further information about plants featured in the show, with audience reaction driven by the screen time.

**Conclusions:**

Natural history films can promote plant awareness, and culturomic tools can be used to assess their impact on the general public, potentially also to inform plant conservation strategies. These are promising findings as we strive to increase public awareness of the value of plant life.

## INTRODUCTION

Our relationship with plants has changed throughout history and now it must change again. We must now work with plants and make the world a little greener, a little wilder. If we do this, our future will be healthier, and safer, and happier. Plants are, after all, our most ancient allies, and together we can make this an even greener planet. **David Attenborough**, closing of *Green Planet*.

Life on Earth depends on plants, and they are essential for human survival. Plant biodiversity needs to be preserved as an unexploited treasury of wild species that, in the future, might provide new sources of food, medicinal products, biofuels or genetic information for improvement of domesticated crops. However, threats to plant biodiversity intensify, with recent predictions suggesting that close to two in five known plant species might be facing extinction (Nic Lughadha *et al.*, 2020) and that plant species not currently useful to humans might be more likely to become extinct (Kress and Krupnick, 2022). This means that native plant resources, potentially holding solutions to key grand challenges faced by humankind, might be lost before even being identified and used. This is to say nothing of the importance of plant biodiversity for ecosystem maintenance in general, where different species are required to promote ecosystem functioning at different locations and times and in different environmental conditions (Isbell *et al.*, 2011). Conserving plant biodiversity in a changing world is therefore vital and requires the concerted effort of scientists and society (Knapp, 2019).

Nonethless, people struggle to recognize the presence and importance of plants owing to a cognitive bias initially described as plant blindness (Wandersee and Schussler, 1999; Schussler, 2001) and recently renamed plant awareness disparity (Parsley, 2020). Unsurprisingly, plant awareness disparity raises challenges for plant conservation. It was suggested to obscure the scale of the extinction crisis for plant species (Halpin and McKim, 2019) and to be a crucial reason for lower public coverage, support and funding dedicated to plant conservation programmes (Balding and Williams, 2016). Indeed, the scale of the problem is significant (Balding and Williams, 2016): US data show that plants received <4% of federal funding for endangered species, despite composing 57% of the endangered species list (Havens *et al.*, 2014); plant community or species research represented only 18% of sampled conservation studies from Spain (Martín-López *et al.*, 2009); and although a huge fraction of illegally and legally traded wildlife are plants and invertebrates, most analyses on global wildlife trade are biased towards vertebrates (Fukushima *et al.*, 2020). With people already suffering from an extinction of experience from all forms of life owing to urban living (Soga and Gaston, 2016) and with the erosion of botanical education (Stroud *et al.*, 2022), these examples highlight the added urgent need to address the conservation bias against plants in order to solve the biodiversity crisis. Increasing plant awareness seems at least part of the solution, because engaged communities that understand and appreciate nature are willing to support its protection actively (Dobson, 2007; Aslin and Lockie, 2013; Dean *et al.*, 2016; Day *et al.*, 2022). Arguably, the time for addressing plant awareness disparity and for promoting plant conservation initiatives has never been better. Recently, we commented on increased public interest in plants as a hobby and as lifestyle elements and the opportunity it offers to develop an appreciation of botany among the wider public (Burke *et al.*, 2022). Here, we address the question: can nature documentaries build on this trend and promote plant awareness, ultimately increasing audience engagement with plant conservation programmes?

Several landmark natural history productions, including *Planet Earth II*, *Blue Planet II* and *Seven Worlds, One Planet*, have raised awareness for presented animal species, although their role in promoting conservation engagement is less well defined because they often contain only limited explicit messaging in this area (Fernández-Bellon and Kane, 2020; Nolan *et al.*, 2022). Nevertheless, these premium nature documentaries provide a means to reach mass audiences, engage them and create lasting impact of a magnitude comparable to that achieved by conservation-focused campaigns, such as ‘world species days’ (Fernández-Bellon and Kane, 2020). The effect of nature documentaries on plant awareness, however, remains largely unexplored, with previous analyses being focused on the featured animals (Fernández-Bellon and Kane, 2020).

We investigated this using the conceptual framework developed by Fernández-Bellon and Kane (2020), here applied to the *Green Planet* show, a high-budget 2022 BBC documentary narrated and presented by Sir David Attenborough. *Green Planet* was broadcast by the BBC in early 2022 and achieved record audiences of >5 million UK viewers per episode (BARB, 2022). Each of the five episodes of *Green Planet* focused on a distinct theme, showcasing diversity of plants from tropical rainforests, aquatic environments, seasonal lands, deserts and urban spaces; and tackling issues such as the dangers of invasive monocultures or deforestation. The show, which premiered at the COP26 summit, was praised by critics for environmental messaging, encouraging plant appreciation and conservation (Bourke, 2022; Cockburn, 2022; Hunt, 2022). The potential of *Green Planet* to counteract plant awareness disparity was also broadly recognized (Guardian, 2022; Pooran, 2022). Therefore, the show provided an ideal springboard for examining the effect of popular nature documentaries on the plant awareness of the audience. To achieve this, we adopted a culturomics approach (i.e. using big data to study human behaviour in the context of conservation; Michel *et al.*, 2011) based on analyses of Google search engine and Wikipedia activities.

## METHODS

### Plant mentions

We identified all plants mentioned in the script of *Green Planet* episodes that fitted into a taxonomic rank of family or lower (*n* = 64). For each plant mentioned in the show, we also recorded the total time on screen (Supplementary Data Table S1A).

### Audience reaction analyses

To evaluate the interest of the audience in portrayed plants, we considered online searches seeking further information as a form of engagement. Google is the most popular search engine, holding >85% of the market share (Johnson, 2022). To obtain data on users’ web interests, we used the Google Trends tool, because it provides straightforward access to these search data without complications related to privacy (Rovetta, 2021) and is being used increasingly for various other types of culturomic studies, also in the context of conservation studies and human–nature interactions (Proulx *et al.*, 2014; Troumbis and Vasios, 2021; Vardi *et al.*, 2021; Troumbis *et al.*, 2022). Google Trends data are normalized: each data point is divided by the total searches of the geography and time range it represents to compare relative popularity, with 100% representing the peak popularity of a searched term. We downloaded weekly Google Trends data for the period between January 2021 (~1 year before release of the first episode, in order to detect potential seasonal effects) and May 2022 (~3 months after the last episode), with UK set as the search geographical area, because that was where *Green Planet* was broadcast in January and February 2022 (recorded in Supplementary Data Table S1B). Search terms corresponding to plant common names used in the *Green Planet* show were used for data extraction. Data for the same search terms in the USA were used as a control (recorded in Supplementary Data Table S1C), to test the internet search behaviours among an English-speaking audience (hence using the same search terms) that had not been exposed to the *Green Planet* show during the investigated period. Searched terms were considered as generating a significant signal (interest) if the peak popularity (100%) occurred within 1 week starting from broadcast of the relevant episode. As an additional control, we extracted the Google Trends data for eight species from a web article about the most intriguing plants from the rainforest (Brusowankin, 2022) that were not mentioned in the *Green Planet* episode dedicated to this biome (Episode 1, ‘Tropical Worlds’, release date 9 January 2021) and investigated whether they generated a signal after the broadcast (recorded in Supplementary Data Table S1D).

We also investigated Wikipedia view patterns associated with pages representing plants mentioned in the show. Wikipedia is the most popular online encyclopedia and can therefore be considered an indicator of public interest in a range of topics (Okoli *et al.*, 2014). The R packages *pageviews* (Keyes and Lewis, 2020) and *AnomalyDetection* (Vallis *et al.*, 2014) were used as previously described by Fernández-Bellon and Kane (2020), with settings allowing a maximum of three anomalies for a page/species over the investigated period (from 1 June 2021 to 30 April 2022). The script is available at: https://github.com/kanead/Green_planet. Species with at least one anomaly date falling within 1 week after release of the episode in which they were mentioned were considered as generating a signal (interest) (recorded in Supplementary Data Table S1E). The 1-week period post-broadcast was selected to enable comparison with weekly search data downloaded from Google Trends, and owing to new episodes of the show being released weekly. We also examined the Wikipedia page view patterns for the control species from tropical rainforest that were not mentioned in the *Green Planet* ‘Tropical Worlds’ episode (recorded in Supplementary Data Table S1F).

## RESULTS AND DISCUSSION

**Fig. 1.**
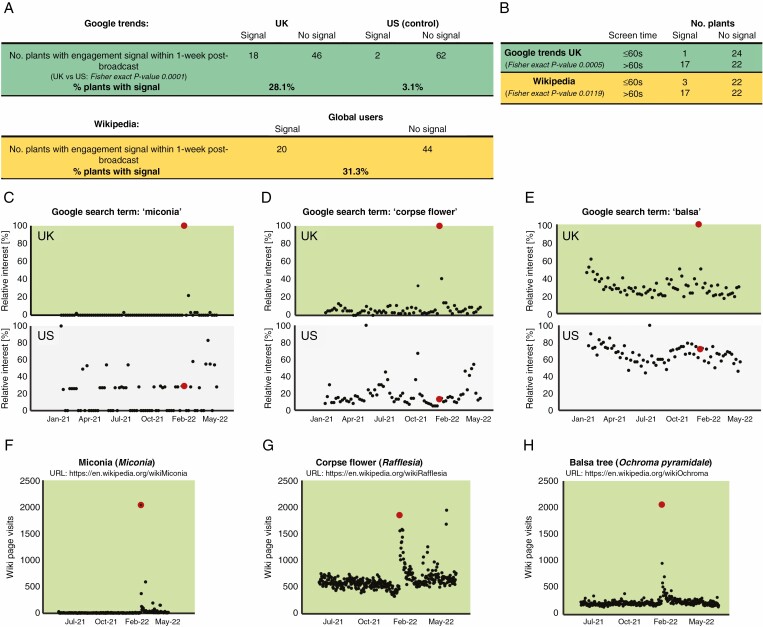
Big data demonstrate impact of *Green Planet*. (A) Number of plants mentioned in *Green Planet* that generated an engagement signal ≤1 week after the broadcast of the relevant episode, based on Google Trends and Wikipedia page visit analyses. The Google Trends data from US web users, who were not exposed to the show, were used as a control. (B) Comparison of engagement signal generated by plants with shorter (≤60 s) and longer (>60 s) screen times. Fisher’s exact *P*-values indicate a significant difference. (C–E) Time series of relative interest of UK and US users (Google search term popularity) for the terms ‘miconia’, ‘corpse flower’ and ‘balsa’, respectively. Red data points correspond to the broadcast date of the *Green Planet* episode in which these plant species were featured (6 February 2022 for miconia and 9 January 2022 for corpse flower and balsa tree). (F–H) Time series of visits to Wikipedia pages representing miconia (*Miconia*), corpse flower (*Rafflesia*) and balsa tree (*Ochroma pyramidale*), respectively. Again, red data points correspond to the broadcast date of the *Green Planet* episode in which these plant species were featured.

The results of the Google Trends analysis indicated a sizeable effect of *Green Planet* on the awareness and interest of viewers in the portrayed plant species (Fig. 1); 28.1% (18 of 64) of search terms representing plants mentioned in the BBC documentary had peak popularity in the UK during the week after broadcast of the relevant episode. In comparison, peak popularity during the same period was detected for only 3.1% (2 of 64) of the same search terms among the control, US-based web users not exposed to the show at the time (Fig. 1A), with Fisher’s exact *P*-value of 0.0001, indicating a significant difference in engagement for information between UK and US users.

This suggests that *Green Planet* stimulated interest in the portrayed plants that extended beyond the broadcast of the show. The Wikipedia search data recapitulated this conclusion, with 31.3% (20 of 64) pages corresponding to plants mentioned in *Green Planet* showing increased visits during the week after the broadcast (Fig. 1A), although direct comparison of US- and UK-based web users was not possible in this case, because the Wikipedia pageviews data are not separated by geography. The positive effect of the *Green Planet* show on audience engagement for information was also highlighted by the fact that although 37.5% (three of eight) plants mentioned in the first episode, ‘Tropical Worlds’, generated an increased audience after broadcast at both Google search and Wikipedia pageviews levels (Supplementary Data Table S1C, E), this was not observed for any of eight control plant species from this biome that were not portrayed in the show (Supplementary Data Table S1D, F).

As anticipated, the plants with longer screen times (>60 s) were more likely to trigger the interest of viewers (Google searches or Wikipedia site visits; Fig. 1B). These impacts of *Green Planet* will probably be leveraged further thanks to popularity of the presenter and narrator, David Attenborough, who spent his almost 70-year-long career being a prominent advocate for nature and wildlife conservation. Opinion leaders, such as Attenborough, have been suggested to have strong potential to promote messaging from nature documentaries (de Lange *et al.*, 2019). Indeed, while promoting the show, David Attenborough put a strong emphasis on the importance of plants (‘… we would starve without plants. And we literally wouldn’t be able to breathe without plants …’), recognized mistakes in our attitudes to plants (‘… a parallel world on which we depend, and which up to now we have largely ignored …’) and highlighted the necessity to protect plant wildlife (‘Over half the population of the world according to the United Nations are urbanised, live in cities, only see cultivated plants and never see a wild community of plants. But that wild community is there, outside urban circumstances normally, and we depend upon it. And we better jolly well care for it.’) (RTE, 2022).

Our analysis reiterated the previous observations for animals (Fernández-Bellon and Kane, 2020; Nolan *et al.*, 2022) and suggested that plant awareness disparity does not prevent the effects that nature documentaries can have on the general public. Although the viewers already showing interest in plants were likely to be overrepresented among the audience of *Green Planet*, the Broadcasters Audience Research Board (barb.co.uk) reported that on average each episode had attracted 5.4 million viewers on first broadcast, corresponding to 10% of the adult population of the UK and indicating broad reach of the show. Moreover, previous analyses commissioned by the BBC performed by the marketing intelligence platform Affinio highlighted that a previous BBC TV series narrated by Sir David Attenborough, *Planet Earth II*, attracted remarkably diverse communities, categorized into interest-based clusters such as ‘UK Current Affairs’, ‘International Current Affairs/Science and Nature’, ‘UK Football Fanatics’, ‘Entertainment TV Junkies’, ‘Grime/Gaming Boys’, ‘YouTube Generation’, ‘Nature Lovers’ and ‘Competition Women’ (Affinio, 2017). It is therefore plausible that the large audience of *Green Planet* also included a broad range of viewer types, especially considering the high volume of media coverage the show received and the multilayered advertising strategy used by the BBC to promote it. The latter included the ‘Welcome to Their World’ campaign, in which Green Park Station tube station was rebranded to Green Planet, with every poster space across the platform filled with dramatic images of plants (BBC Creative, 2022), in addition to a free augmented reality *Green Planet* experience in London (https://thegreenplanetexperience.co.uk/) and 4K DeepScreen technology animation displayed at Piccadilly Circus (BBC, 2022). The *Green Planet* show was therefore likely to reach and make an impact on even those members of the public with low awareness of plants and their roles in our lives.

What do our findings mean for plant biodiversity and our efforts to protect it? Can nature documentaries not only increase interest in plant species but also promote awareness of plant conservation issues? A recent study has shown that audiences of natural history documentaries engage less with depictions of conservation topics than with the animal species shown on screen (Nolan *et al.*, 2022). Perhaps a species-focused approach, whereby charismatic species provide powerful examples of crucial problems faced by plant biodiversity.

Can achieve this? Miconia (*Miconia calvescens*), described in the last episode of *Green Planet*, is a plant that can form a self-sustaining dense monoculture that now stifles native Hawaiian vegetation. The audience searched for more information on miconia after the broadcast (Fig. 1C, F), in this way becoming more aware of dangers of invasive species in general. Potentially, big data can also be a useful source of information for conservation programmes. For example, they can aid identification of the plants that triggered a strong response in the real-life audiences, causing the viewers to search for more information, which can then be used as ambassadors for plant biodiversity and conservation actions. As argued by Thorogood (2020), such striking, unusual species can be the first motifs that awaken fascination with plants. This could lead later to increased commitment to conservation issues. A good example of this is the corpse flower, or rafflesia, presented in the first episode of the show, which elicited a strong response in viewers (Fig. 1D, G). *Rafflesia* is a genus of parasitic plants with the biggest flowers on Earth, spanning 1 m, and its common name, corpse flower, is attributable to its resemblance to a decaying cadaver and emitted odour of rotten flesh. Owing to these unforgettable characteristics, rafflesia is sometimes called the giant panda of plant world (Molina *et al.*, 2021) and is increasingly considered a Malayan flagship species, representing rare flora of Indonesia threatened by anthropogenic activities (Mursidawati and Wicaksono, 2020). The robust response of the audience of *Green Planet* to this plant confirms that it is indeed a suitable candidate for this role.

Another species presented in the first episode was the balsa tree (*Ochroma pyramidale*), which grows at an incredibly rapid rate and has a unique, very light wood structure, making balsawood the lightest and softest timber used commercially for many specific applications. For these reasons, balsa trees are grown in dense plantations and therefore the species is far from being endangered. However, balsa provides a spectacular example of how plants native to tropical rainforest, the treasure chest of plant biodiversity that needs to be protected, can be incredibly useful to humans. Balsa spiked strong interest among the audience of the *Green Planet* show (Fig. 1E, H), which suggests that it can be also used as an ambassador species representing concepts related to plant biodiversity or conservation.

Compared with animals, plants are not frequently used as flagships, and plant flagship species generally receive less exposure compared with representatives of mammals and birds (Clucas *et al.*, 2008). Nevertheless, the use of plant flagship and charismatic species is attracting increasing interest as a tool for counteracting plant awareness disparity (Pany and Heidinger, 2017), for biodiversity conservation (e.g. Hall *et al.*, 2011; Qian *et al.*, 2020), for ecotourism (Hall *et al.*, 2011; Setiawan, 2017; Agius *et al.*, 2018) or, recently, in the more specific context of trade of medicinal ingredients from wild plants (Schindler *et al.*, 2022). Several approaches aimed at identification of suitable plant flagship species have been proposed (Pany and Heidinger, 2017; Qian *et al.*, 2020). Our analysis suggests that big data mining can also contribute to selection of charismatic species for conservation programmes by measuring how mass audiences respond to different plants and by identifying those that elicit the strongest interest of the public.

## CONCLUSIONS

In conclusion, *Green Planet* demonstrated the potential of natural history documentaries to drive mass audience engagement and increase awareness of presented plant species. In the broader context, we suggest that big data analyses could be a useful approach to probe how audiences respond to plant species presented in natural history films or other forms of mainstream media and can inform plant conservation strategies.

## SUPPLEMENTARY DATA

Supplementary data are available at *Annals of Botany* online and consist of the following.

Table S1: Weekly Google Trends data and Wikipedia page views anomaly dates extracted for plants mentioned in the *Green Planet* show, and the control plant species group.

mcac149_suppl_Supplementary_Data
